# Differentially Expressed Potassium Channels Are Associated with Function of Human Effector Memory CD8^+^ T Cells

**DOI:** 10.3389/fimmu.2017.00859

**Published:** 2017-07-24

**Authors:** Ji Hyun Sim, Kyung Soo Kim, Hyoungjun Park, Kyung-Jin Kim, Haiyue Lin, Tae-Joo Kim, Hyun Mu Shin, Gwanghun Kim, Dong-Sup Lee, Chan-Wook Park, Dong Hun Lee, Insoo Kang, Sung Joon Kim, Chung-Hyun Cho, Junsang Doh, Hang-Rae Kim

**Affiliations:** ^1^Department of Anatomy and Cell Biology, Seoul National University College of Medicine, Seoul, South Korea; ^2^Department of Biomedical Sciences, Seoul National University College of Medicine, Seoul, South Korea; ^3^Department of Physiology, Seoul National University College of Medicine, Seoul, South Korea; ^4^Department of Mechanical Engineering, Pohang University of Science and Technology, Pohang, South Korea; ^5^Department of Pharmacology, Seoul National University College of Medicine, Seoul, South Korea; ^6^BK21Plus Biomedical Science Project, Seoul National University College of Medicine, Seoul, South Korea; ^7^Medical Research Institute, Seoul National University College of Medicine, Seoul, South Korea; ^8^Department of Obstetrics and Gynecology, Seoul National University College of Medicine, Seoul, South Korea; ^9^Department of Dermatology, Seoul National University College of Medicine, Seoul, South Korea; ^10^Department of Internal Medicine, Section of Rheumatology, Yale University School of Medicine, New Haven, CT, United States

**Keywords:** human effector memory CD8^+^ T cells, interleukin-7Rα^low^ effector memory CD8^+^ T cells, calcium-activated potassium channel KCa3.1, voltage-gated potassium channel Kv1.3, T-cell motility, transendothelial migration

## Abstract

The voltage-gated potassium channel, Kv1.3, and the Ca^2+^-activated potassium channel, KCa3.1, regulate membrane potentials in T cells, thereby controlling T cell activation and cytokine production. However, little is known about the expression and function of potassium channels in human effector memory (EM) CD8^+^ T cells that can be further divided into functionally distinct subsets based on the expression of the interleukin (IL)-7 receptor alpha (IL-7Rα) chain. Herein, we investigated the functional expression and roles of Kv1.3 and KCa3.1 in EM CD8^+^ T cells that express high or low levels of the IL-7 receptor alpha chain (IL-7Rα^high^ and IL-7Rα^low^, respectively). In contrast to the significant activity of Kv1.3 and KCa3.1 in IL-7Rα^high^ EM CD8^+^ T cells, IL-7Rα^low^ EM CD8^+^ T cells showed lower expression of Kv1.3 and insignificant expression of KCa3.1. Kv1.3 was involved in the modulation of cell proliferation and IL-2 production, whereas KCa3.1 affected the motility of EM CD8^+^ T cells. The lower motility of IL-7Rα^low^ EM CD8^+^ T cells was demonstrated using transendothelial migration and motility assays with intercellular adhesion molecule 1- and/or chemokine stromal cell-derived factor-1α-coated surfaces. Consistent with the lower migration property, IL-7Rα^low^ EM CD8^+^ T cells were found less frequently in human skin. Stimulating IL-7Rα^low^ EM CD8^+^ T cells with IL-2 or IL-15 increased their motility and recovery of KCa3.1 activity. Our findings demonstrate that Kv1.3 and KCa3.1 are differentially involved in the functions of EM CD8^+^ T cells. The weak expression of potassium channels in IL-7Rα^low^ EM CD8^+^ T cells can be revived by stimulation with IL-2 or IL-15, which restores the associated functions. This study suggests that IL-7Rα^high^ EM CD8^+^ T cells with functional potassium channels may serve as a reservoir for effector CD8^+^ T cells during peripheral inflammation.

## Introduction

The voltage-gated potassium channel, Kv1.3 (1), and the Ca^2+^-activated potassium channel, KCa3.1 (also known as SK4) (2), play crucial roles in T cell activation and function (3–8). Activation of T cells occurs upon encountering their cognate antigens and requires a sustained or oscillatory increase in intracellular Ca^2+^ concentration ([Ca^2+^]_i_). The increased [Ca^2+^]_i_ is initiated by IP_3_-mediated Ca^2+^ release from the endoplasmic reticulum storage site and maintained by Ca^2+^ entry *via* calcium release-activated calcium (CRAC) channels in the plasma membrane (9). The increase in [Ca^2+^]_i_ leads to the activation of KCa3.1. CRAC channel activation depolarizes the cells, subsequently activating Kv1.3. The negative membrane potential maintained by activation of the potassium channels provides an electrical driving force for the influx of Ca^2+^, which is crucial for T cell activation (7).

An electrophysiological analysis of Kv1.3 and KCa3.1 in activated effector memory (EM) CD8^+^ T cells was reported previously (10). However, a recent retrospective examination based on the current classification of human memory CD8^+^ T cell subsets leads us to revisit the expression and activities of the potassium channels in the CD8^+^ T cell subsets and their physiological consequences. As the expression of CCR7 and CD45RA memory markers on CD8^+^ T cells change upon T cell receptor (TCR) stimulation *in vitro* (11, 12), purification of memory CD8^+^ T cell subsets should be performed prior to stimulation.

Previously, we identified two unique subsets of human EM CD8^+^ T cells (CCR7^−^CD45RA^+/−^) that express high and low levels of the interleukin (IL)-7 receptor alpha chain (IL-7Rα^high^ and IL-7Rα^low^, respectively) in the peripheral blood (13). Compared to IL-7Rα^high^ EM CD8^+^ T cells, IL-7Rα^low^ EM CD8^+^ T cells are largely antigen-experienced (CD27^−^CD28^−^) cells that show increased expression of cytotoxic molecules, such as perforin and granzyme B, and defective proliferation upon TCR stimulation with anti-CD3/CD28 antibodies (Abs) (13). IL-7Rα^low^ EM CD8^+^ T cells show increased frequency with aging (13) and in patients with lupus (14). Additionally, such cells have defects in proliferation (13). Thus, the classification of human EM CD8^+^ T cell subsets based on IL-7Rα expression might be more descriptive of the function of EM CD8^+^ T cells than the previous classification method based on the expression of the chemokine receptors CCR7 and CD45RA (15).

Upon TCR stimulation, these IL-7Rα^low^ EM CD8^+^ T cells displayed impaired proliferation (13), inferring the possibility that Ca^2+^ signaling and, in particular, potassium channels may be involved in signaling pathway. Accordingly, we analyzed the Ca^2+^ influx and investigated whether Kv3.1 and KCa3.1 show different activities in the EM CD8^+^ T cell subsets IL-7Rα^high^ and IL-7Rα^low^ and examined the roles of Kv3.1 and KCa3.1 using pharmacologically specific inhibitors in EM CD8^+^ T cell subsets. We found that the potassium channels in the EM CD8^+^ T cell subsets do differentially regulate their functions such as proliferation, cytokine production, and motility.

## Materials and Methods

### Human Subjects

This work was approved by the Institutional Review Board of Seoul National University Hospital (# 0905-014-280). Peripheral blood was obtained from healthy volunteers who were taking no immunosuppressive drugs and had no diseases that could potentially affect the immune system such as autoimmunity, infections, and malignancies (13). Skin specimens (5 mm diameter) were obtained from a patient who had moderate atopic dermatitis with chronic lesional and non-lesional skin. Written informed consent was obtained from all subjects according to the Declaration of Helsinki.

### Flow Cytometric Analysis and Cell Sorting

Peripheral blood mononuclear cells (PBMCs) in heparinized peripheral blood were isolated using a Ficoll-Histopaque gradient (1.077 g/mL; GE Healthcare Biosciences, Piscataway, NJ, USA). The following anti-human Abs were used for flow cytometry staining: allophycocyanin-anti-CD8, phycoerythrin-anti-CCR7 (all from BD Biosciences, San Jose, CA, USA), and eFluor^®^ 450NC-anti-IL-7Rα (CD127) (eBioscience, San Diego, CA, USA).

Fluorescence-activated cell sorter (FACS) staining was performed as described (13). Staining intracellular molecules was performed after permeabilization using a Cytofix/Cytoperm kit (BD Biosciences). The stained cells were analyzed on a BD LSRII^®^ (BD Biosciences) with FACSDiva software, and the data were analyzed using FlowJo^®^ software (TreeStar, Ashland, OR, USA).

CD8^+^ T cells were enriched from PBMCs using the Human CD8^+^ T Cell Isolation kit (Miltenyi Biotec) and then stained with Abs and sorted into naïve (CCR7^+^CD45RA^+^), IL-7Rα^high^, and IL-7Rα^low^ EM (CCR7^−^CD45RA^+/−^) CD8^+^ T cells using a BD FACSAria^®^ (BD Biosciences).

### *In Vitro* Cell Culture

Sorted CD8^+^ T cell subsets were cultured in RPMI 1640 media containing 10% fetal bovine serum (FBS) and 1% penicillin/streptomycin (i.e., RPMI 1640 complete media; all reagents from Life Technologies, Carlsbad, CA, USA). To reverse CD8^+^ T cells by IL-2 stimulation, freshly sorted CD8^+^ T cell subsets were incubated for 5 days with anti-CD3 (HIT3a, 10 µg/mL, plate-bound)/CD28 (28.2, 5 µg/mL, soluble) Abs (both from eBioscience) in the presence of recombinant human IL-2 (20 IU/mL, R&D Systems, Minneapolis, MN, USA) and then stimulated for 10 days in the presence of IL-2 alone with media changes every 3 days with IL-2 replenishment (16) (Sim et al., manuscript submitted).

To stimulate CD8^+^ T-cell subsets with cytokines, cells were stimulated for 3 days with anti-CD3/CD28 Abs in the presence or absence of IL-2 (20 IU/mL), IL-15 (5 ng/mL; PeproTech, Rocky Hill, NJ, USA), or IL-4 (5 ng/mL; PeproTech).

For cytokine measurement, sorted CD8^+^ T cell subsets were stimulated for 24 h with anti-CD3/CD28 Abs. Culture supernatants were analyzed for cytokines using a MILLIPLEX^®^ MAP kit (EMD Millipore, Billerica, MA, USA) according to the manufacturer’s instructions.

For measuring cell proliferation, sorted CD8^+^ T cell subsets were labeled with 0.5 µg/mL carboxyfluorescein diacetate (CFSE, Life Technologies) and incubated for 6 days with anti-CD3/CD28 Abs. Cell proliferation was analyzed using a flow cytometer.

Cells were incubated with 50 µM 1-ethyl-2-benzimidazolinone (1-EBIO) or with 5 µM (1-[(2-chlorophenyl)diphenylmethyl]-1H-pyrazole, TRAM-34) to activate or inhibit the KCa3.1 channel, respectively, or with margatoxin (5 nM) (all from Sigma-Aldrich, St. Louis, MO, USA) to inhibit the Kv1.3 channel during TCR stimulation with anti-CD3/CD28 Abs.

### Electrophysiology

Electrophysiological measurements were performed using the conventional whole-cell recording mode at room temperature. Patch pipettes with a free-tip resistance of 5–7 MΩ were connected to the head stage of an Axopatch 200B patch-clamp amplifier (Molecular Devices, Sunnyvale, CA, USA). pCLAMP software v9.0 and Digidata-1332A (Molecular Devices) were used to acquire data and apply command pulses. Cells were transferred into a bath (~0.15 mL) mounted on the stage of an inverted microscope (IX50; Olympus, Tokyo, Japan) and perfused with HEPES-buffered normal Tyrode (NT) solution at 5 mL/min. The NT external solution was composed of 145 mM NaCl, 5 mM glucose, 3.6 mM KCl, 1.3 mM CaCl_2_, 1 mM MgCl_2_, and 10 mM HEPES (adjusted to pH 7.4 with NaOH). For whole-cell patch clamp analysis of Kv1.3 and KCa3.1 channels, the pipette solution contained 140 mM KCl, 5 mM NaCl, 2 mM MgATP, 5 mM EGTA, 4.37 mM CaCl_2_, 0.5 mM MgCl_2_, and 10 mM HEPES (pH 7.2) with free Ca^2+^ concentration fixed at 1 µM. Phosphatidylinositol 3-kinase (PI3K) inhibitor (10 µM LY294002, Sigma-Aldrich) was included in the intracellular pipette solution in several experiments as indicated.

**Figure 1:**
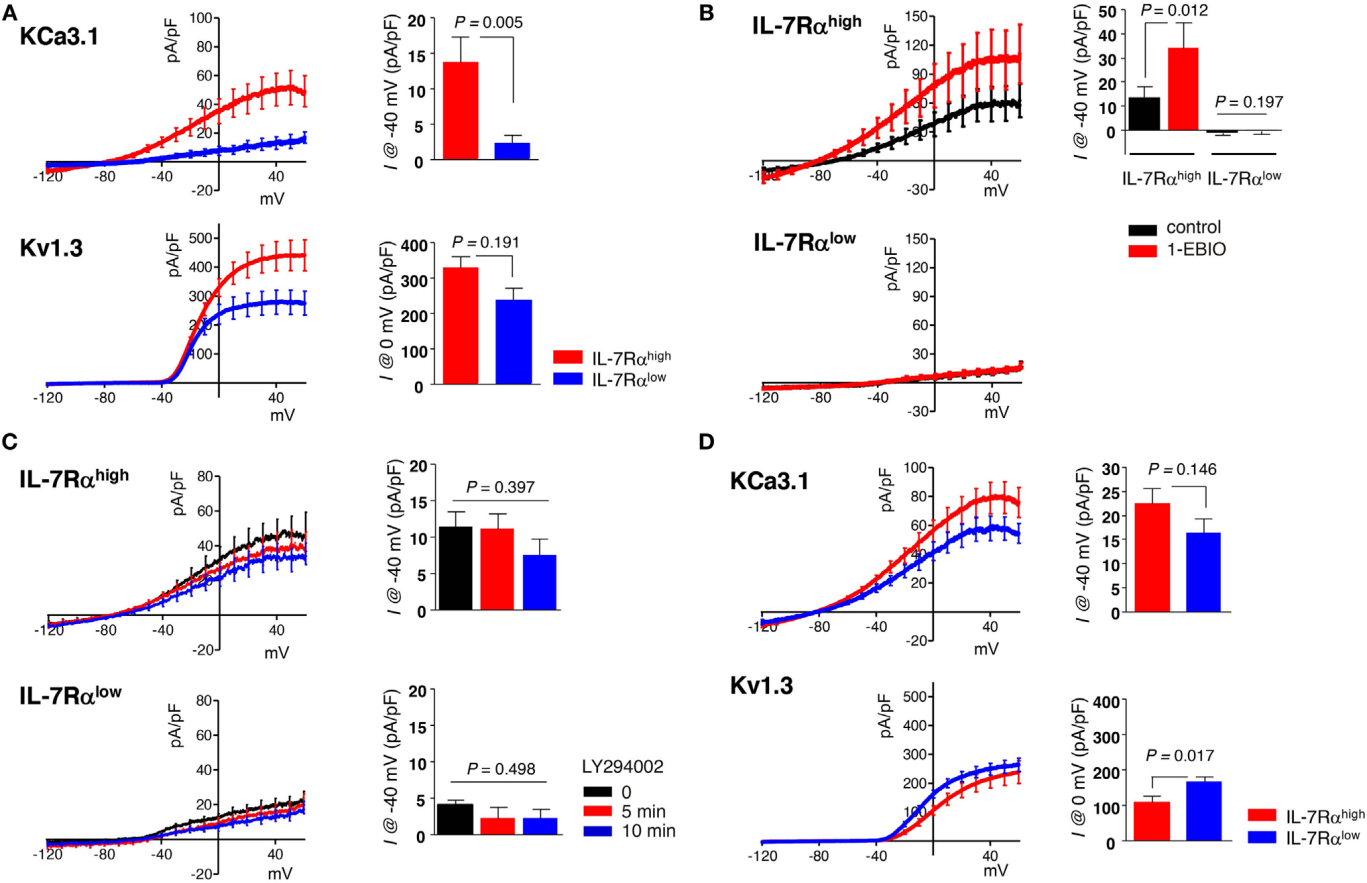
Human effector memory (EM) CD8^+^ T cell subsets differentially express Kv1.3 and KCa3.1. **(A–C)** Peripheral blood mononuclear cells from healthy individuals were stained with antibodies (Abs) to CD8, C-C chemokine receptor type 7 (CCR7), and interleukin (IL)-7Rα and sorted into IL-7Rα^high^ and IL-7Rα^low^ subsets of EM CD8^+^ T cell using a BD FACSAria^®^. **(A)** Freshly sorted CD8^+^ T cell subsets were stimulated for 24 h with anti-CD3/CD28 Abs, and their KCa3.1 (IL-7Rα^high^, *n* = 23; IL-7Rα^low^, *n* = 23) and Kv1.3 (IL-7Rα^high^, *n* = 18; IL-7Rα^low^, *n* = 19) current components underlying the total current–voltage (I–V) curve were measured as follows: (i) the maximum inactivation of Kv1.3 was induced using depolarized holding voltage (−10 mV); (ii) a reverse ramp-like pulse from 60 to −120 mV was applied; and (iii) the obtained I–V curve for KCa3.1 was subtracted from the initial I–V curve obtained using a forward ramp-like pulse in the whole-cell configuration for the Kv1.3 current. **(B)** KCa3.1 was activated by subsequently adding 50 µM 1-ethyl-2-benzimidazolinone (1-EBIO) to verify the presence of functional KCa3.1 in IL-7Rα^high^ (*n* = 8) and IL-7Rα^low^ (*n* = 10) EM CD8^+^ T cells. **(C)** Cells were treated with a phosphatidylinositol 3-kinase inhibitor (10 µM LY294002) in IL-7Rα^high^ (*n* = 7) and IL-7Rα^low^ (*n* = 8) EM CD8^+^ T cells. **(D)** The current components of KCa3.1 (IL-7Rα^high^, *n* = 17; IL-7Rα^low^, *n* = 16) and Kv1.3 (IL-7Rα^high^, *n* = 18; IL-7Rα^low^, *n* = 19) were measured in IL-2 reversed IL-7Rα^high^ and IL-7Rα^low^ EM CD8^+^ T cells. The results were obtained by combining data from two independent experiments using two different donors. Bars and error bars represent the mean ± SEM **(A–D)**, and *p*-values were obtained using the two-tailed Student’s *t*-test **(A,B,D)** or the analysis of variance followed by Tukey’s *post hoc* subgroup analysis **(C)**.

Current–voltage (I–V) curves reflecting the functional expression of Kv1.3 and KCa3.1 channels were obtained using ramp-like pulse protocols. Using a whole-cell configuration, the total membrane conductance was initially obtained by applying a depolarizing ramp pulse (from −120 to 60 mV, held at −60 mV). Subsequently, in the same cell, a hyperpolarizing ramp pulse from 60 to −120 mV was applied with −10 mV of depolarized holding voltage to induce inactivation of Kv1.3. The remaining non-inactivating conductance recorded with 1 µM of free Ca^2+^ concentration was regarded as the KCa3.1 current based on the characteristic weak inwardly rectifying I/V curve and the activation by KCa3.1 activator (50 µM 1-EBIO, Figures 1A,B). Digital subtraction of the KCa3.1 I/V curve from the initial total I/V curve provided the Kv1.3 current, demonstrating the characteristic outwardly rectifying voltage-dependent property (Figure 1A, lower panel).

### Migration Assay

For migration assays (17), a flat polyurethane acrylate (PUA) surface on glass coverslips was coated with recombinant human intercellular adhesion molecule 1 (ICAM-1) Fc chimera (ICAM-1, R&D Systems) and/or recombinant human stromal cell-derived factor (SDF)-1α (CXCL12, PeproTech) by incubating ICAM-1 (10 µg/mL) in the absence or presence of SDF-1α (2 µg/mL) in PBS for 3 h at 37°C after a brief (~60 s) air plasma treatment (100 W; Femto Science, Hwaseong, South Korea). ICAM-1 was used because it binds to integrin LFA-1 on the T cell membrane and causes T cell attachment, polarization, and random migration (18).

For migration assays, CD8^+^ T cell suspensions in RPMI complete media were added on flat PUA surfaces in the Chamlide magnetic chamber (Live Cell Instrument, Seoul, South Korea) and maintained in a cell culture incubator at 37°C for 1 h before starting the migration experiments. Ultra-low-melt agarose (USB, Cleveland, OH, USA) at a final concentration of 1% was added to RPMI media to minimize convection during live cell imaging. Unless otherwise noted, we used freshly sorted cells without TCR stimulation in cell migration experiments.

For KCa3.1 channel inhibition, cells were treated with 5 µM TRAM-34 for 2 h before starting the migration experiments.

### Live Cell Imaging and Data Analysis

A modified Zeiss Axio Observer Z1 epifluorescence microscope (Jena, Germany) with a 40× (numerical aperture = 1.3; Plan-Neofluar) objective lens and a Roper Scientific CoolSnap HQ CCD camera (Photometrics, Tucson, AZ, USA) were used for imaging (17). The CD8^+^ T cell-seeded surfaces were mounted on the microscope stage equipped with a Chamlide TC incubator system (Live Cell Instrument) and maintained at 37°C and 5% CO_2_ for live cell imaging. Time-lapse microscopy was initiated with differential interference contrast images recorded at 15 s intervals for 10 min. The trajectory of the CD8^+^ T cells was analyzed using the manual tracking plugin of ImageJ (http://imagej.nih.gov/ij/, National Institute of Health, Bethesda, MD, USA), and quantitative analysis was performed with a custom built program on Matlab (MathWorks, Natick, MA, USA).

### Transendothelial Migration Assay

For transendothelial migration analysis (19), human umbilical vein endothelial cells (HUVECs, Lonza, Basel, Switzerland) were grown on 8-µm microporous membranes in transwell chambers (Life Technologies) under EGM™-2 (Lonza) plus FBS 5% for 48 h. CD8^+^ T cell subsets (1.5 × 10^5^ cells/well) were added above the HUVEC monolayers on the filters. Then, the transwells were placed into a 24-well plate containing 100 ng/mL SDF-1α as a chemoattractant. After 20 h of incubation, the number of cells that had migrated into the lower chambers or underneath the upper transwells was determined by counting the number of cells. The results are expressed as the number of cells that migrated across the filter.

### Immunofluorescence Staining and Confocal Microscopy

Human skin specimens were snap frozen in liquid nitrogen and stored at −80°C. OCT compound (Tissue-Tek^®^, Sakura Finetek USA, Torrance, CA, USA) was used in embedding tissues for frozen section. Tissue sections (7 µm) were fixed in 4% paraformaldehyde, blocked with a blocking buffer (5% goat serum and 5% BSA in PBS) for 30 min at room temperature, and stained with purified anti-human perforin Ab (Mabtech, Nacka Strand, Sweden), Alexa647-conjugated anti-human CD8 Ab (BD Biosciences), biotin-conjugated anti-human IL-7Rα Ab (13-1278, eBioscience), Streptavidin-Cy3 (Life Technologies), and Alexa488-conjugated anti-mouse IgG Ab (Life Technologies) at 4°C overnight. After mounting with DAPI-containing media (AR-6501-01, ImmunoBioScience, Mukilteo, WA, USA), the stained tissue sections were analyzed using a confocal microscopy system (A1, Nikon, Tokyo, Japan) and NIS-Elements viewer (Nikon).

For calculating the number of cells in CD8^+^ T cell subsets, IL-7Rα^+^ and perforin^+^ CD8^+^ T cells were counted for each tissue section, with at least four fields including the epidermis and dermis. Cells were enumerated in the tissue fields of the epidermis and dermis by manual counting.

### Statistical Analysis

All data are expressed as mean ± SEM. Data were compared using one-way analysis of variance followed by Tukey’s *post hoc* test, two-tailed Student’s *t*-test, Wilcoxon matched pairs test, and Mann–Whitney *U* test. *p*-Values <0.05 were considered to indicate significance. All statistical analyses were performed using GraphPad Prism 6.01 (GraphPad Software, La Jolla, CA, USA).

## Results

### Differential Expression of Kv1.3 and KCa3.1 in EM CD8^+^ T Cell Subsets

We conducted whole-cell patch clamp analysis and dissected the membrane conductance for KCa3.1 and Kv1.3 based on the specific inhibitor and biophysical properties (see Subsection “Electrophysiology” in Section “Materials and Methods”) in IL-7Rα^high^ and IL-7Rα^low^ EM CD8^+^ T cell subsets, derived from PBMCs of healthy individuals, after TCR stimulation with anti-CD3/CD28 Abs for 24 h derived from PBMCs of healthy individuals. In contrast to the significant activity of KCa3.1 in IL-7Rα^high^ EM CD8^+^ T cells (mean ± SEM, 13.66 ± 5.55 pA/pF), the corresponding current was almost absent in IL-7Rα^low^ EM CD8^+^ T cells (2.17 ± 1.26 pA/pF). The Kv1.3 current was observed in both cell groups, and the amplitudes appeared to be higher in IL-7Rα^high^ than IL-7Rα^low^ EM CD8^+^ T cells (329.55 ± 30.61 versus 237.74 ± 33.81 pA/pF), although the difference was not statistically significant (Figure 1A). To further confirm the large difference in KCa3.1 activity, we compared the effects of KCa3.1 activator (1-EBIO, 50 µM). In contrast to the consistent increase of KCa3.1 current in IL-7Rα^high^ EM CD8^+^ T cells (control; 13.52 ± 4.46 pA/pF, 1-EBIO; 34.08 ± 10.41 pA/pF), no significant increase was observed in the IL-7Rα^low^ EM CD8^+^ T cells in the presence of 1-EBIO (Figure 1B). These results suggest that the lack of KCa3.1 current was due to the insignificant expression of functional KCa3.1 in the cell membrane. Meanwhile, the expression of Kv1.3 and KCa3.1 channels at the transcriptional level were comparable between IL-7Rα^high^ and IL-7Rα^low^ EM CD8^+^ T cells (Figure S2A in Supplementary Material). Thus, it is presumed that the posttranslational regulation of potassium channel proteins could lead to the functional difference of ionic currents (20, 21). Phosphatidylinositol 3-kinase-dependent signaling positively regulates KCa3.1 activity (22). However, treatment with PI3K inhibitor LY294002 did not affect the conductance of KCa3.1 in IL-7Rα^high^ EM CD8^+^ T cells (0 min; 11.35 ± 2.17 pA/pF, 5 min; 11.09 ± 2.15 pA/pF, 10 min; 7.49 ± 2.24 pA/pF) (Figure 1C). We also compared the changes in [Ca^2+^]_i_ upon TCR stimulation between the two groups. We found that the relatively slow increase in [Ca^2+^]_i_ reflecting Ca^2+^ influx was reduced in IL-7Rα^low^ EM CD8^+^ T cells (Figure S1 in Supplementary Material), suggesting that the potassium channel activities may be involved in the relatively low Ca^2+^ signal.

Clonal anergy of T cells is reversed by IL-2 stimulation (i.e., IL-2 reversal) (16). In fact, IL-2-reversed IL-7Rα^low^ EM CD8^+^ T cells developed into functionally competent cells in terms of cell proliferation and IL-2 production (Sim et al., manuscript submitted). Interestingly, IL-2 reversal rescued the KCa3.1 current in the IL-7Rα^low^ EM CD8^+^ T cells (16.41 ± 2.80 pA/pF). In addition, the Kv1.3 current became slightly larger in the IL-2-reversed IL-7Rα^low^ EM CD8^+^ T cells than in the IL-2-reversed IL-7Rα^high^ EM CD8^+^ T cells (162.46 ± 13.39 versus 106.32 ± 16.09 pA/pF) (Figure 1D).

### Requirement for Kv1.3 in EM CD8^+^ T Cell Proliferation and IL-2 Production

**Figure 2:**
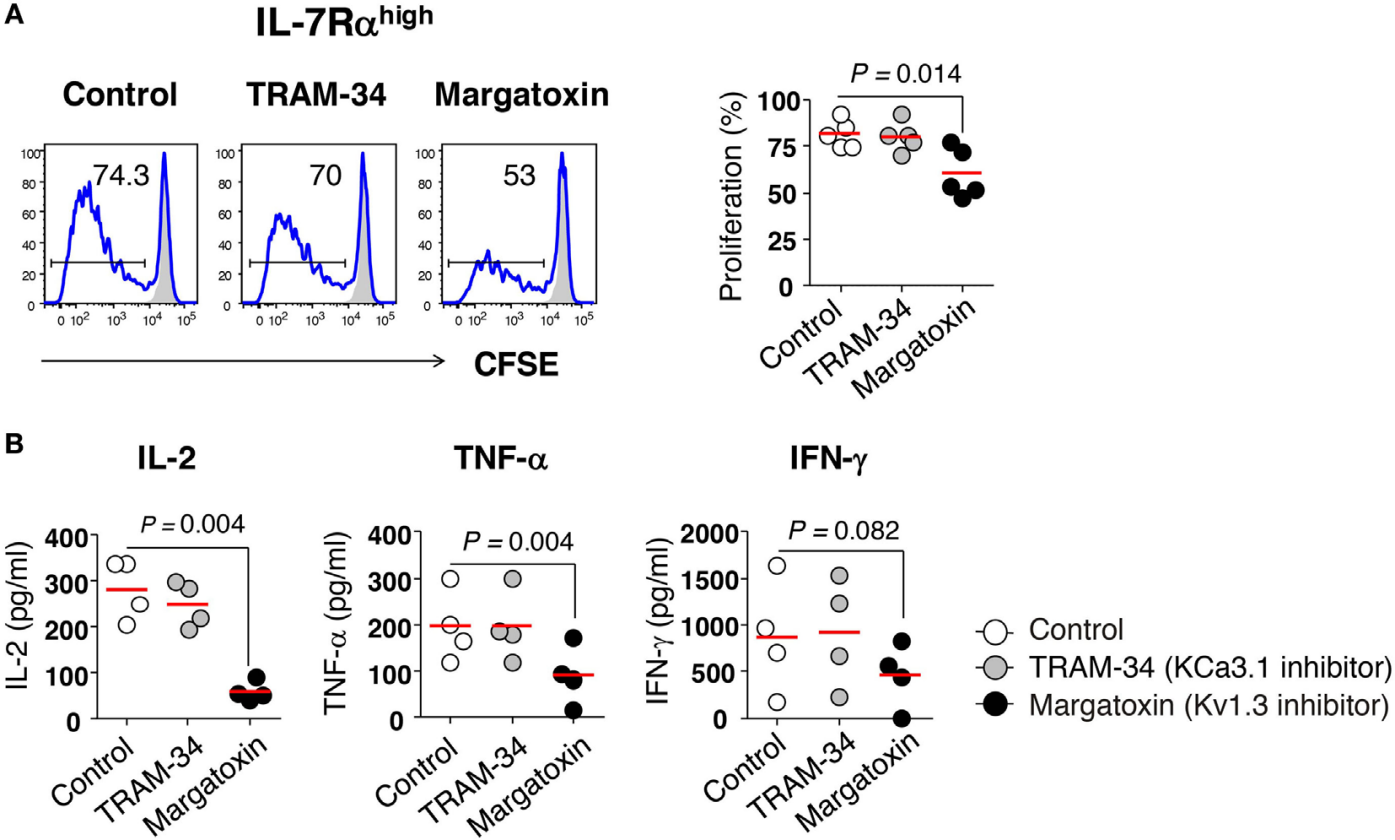
Requirement of Kv1.3 for effector memory (EM) CD8^+^ T cell proliferation and interleukin (IL)-2 production. **(A)** Freshly sorted IL-7Rα^high^ EM CD8^+^ T cells were labeled with carboxyfluorescein diacetate (CFSE) and stimulated for 6 days with anti-CD3/CD28 antibodies (Abs) in the presence or absence of potassium channel inhibitors such as TRAM-34 (KCa3.1 inhibitor, 5 µM) and margatoxin (Kv1.3 inhibitor, 5 nM), and their proliferation was measured by flow cytometry. Representative histograms and a quantification graph showing proliferating cells are shown. **(B)** Quantification of cytokines in culture supernatants from IL-7Rα^high^ EM CD8^+^ T cells that were stimulated for 24 h with anti-CD3/CD28 Abs in the presence or absence of potassium channel inhibitors using a multiplex cytokine assay. Bars indicate the mean. The results are representative data from two or three independent experiments. Bars represent the mean, and *p*-values were obtained using the paired two-tailed Student’s *t*-test.

We investigated the role of potassium channels in T cell proliferation and cytokine production using specific inhibitors for KCa3.1 (TRAM-34) and Kv1.3 (margatoxin). Margatoxin treatment significantly, although not completely, suppressed the proliferation of IL-7Rα^high^ EM CD8^+^ T cells (Figure 2A) and completely inhibited the secretion of IL-2 and TNF-α, but not IFN-γ in IL-7Rα^high^ EM CD8^+^ T cells upon TCR stimulation with anti-CD3/CD28 Abs (Figure 2B). TRAM-34 treatment had no effect on cytokine production or cell proliferation in IL-7Rα^high^ EM CD8^+^ T cells (Figures 2A,B).

### KCa3.1 Mediates the Motility of EM CD8^+^ T Cells

The importance of KCa3.1 in cell migration has been highlighted by its expression patterns in migrating cells including immune cells (23). In migrating human T cells, KCa3.1 is localized in the uropod rear portion (24). Additionally, adenosine suppresses the motility of fully activated T cells with phytohemagglutinin or anti-CD3/CD28 Abs for 72−96 h *via* inhibition of KCa3.1 channel activity (25).

**Figure 3:**
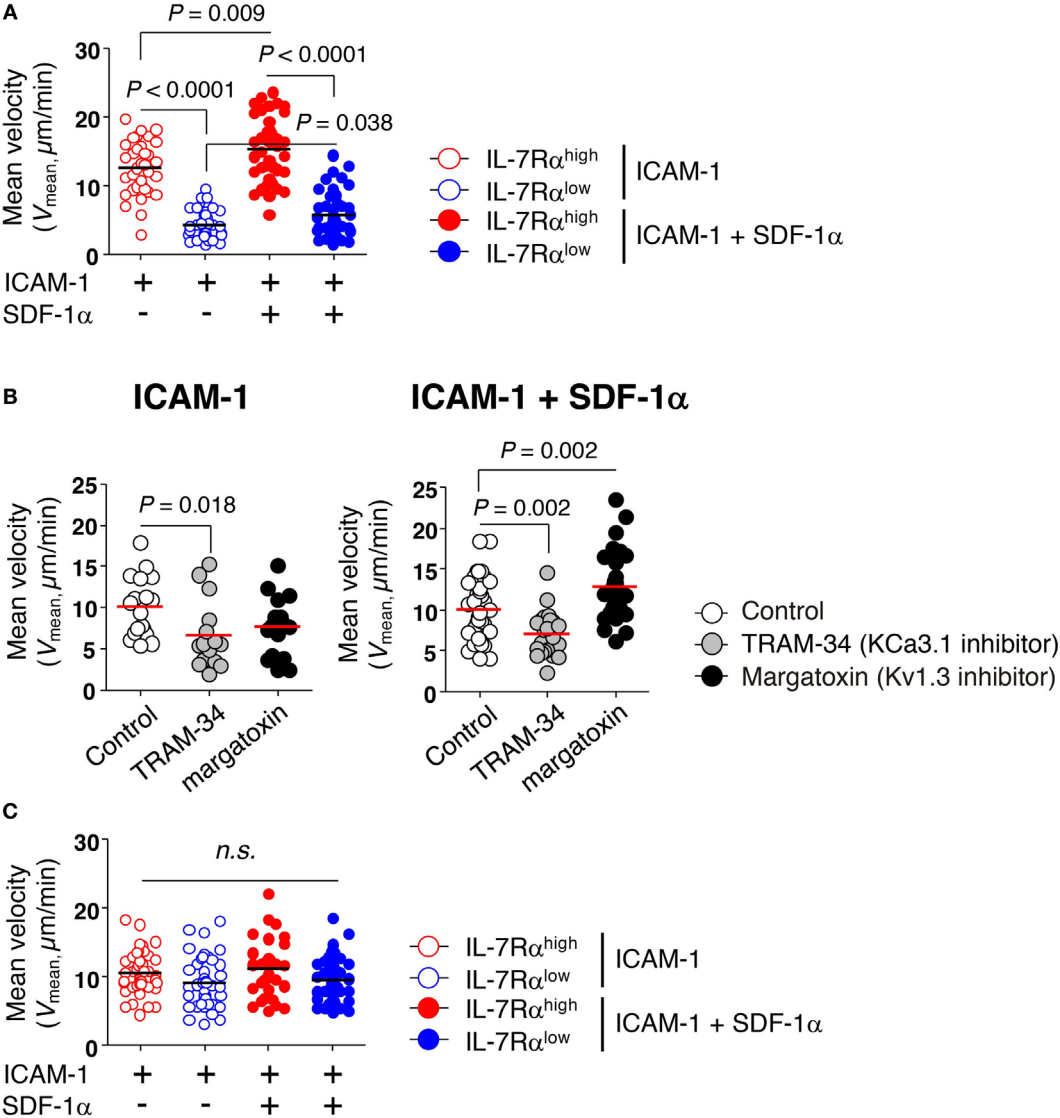
KCa3.1 mediates the motility of effector memory (EM) CD8^+^ T cells. The migration of EM CD8^+^ T cells under agarose gel confinement was recorded by time-lapse microscopy (objective 40×; Zeiss Axio Observer Z1; numerical aperture = 1.3; Plan-Neofluar) and analyzed using ImageJ and Metlab. Flat polyurethane acrylate (PUA) surfaces were coated with 10 µg/mL intercellular adhesion molecule 1 (ICAM-1) and/or 2 µg/mL stromal cell-derived factor (SDF)-1α. **(A)** The effect of ICAM-1 and/or SDF-1α on the mean velocity (*V*_mean_) of interleukin (IL)-7Rα^high^ and IL-7Rα^low^ EM CD8^+^ T cells on flat PUA surfaces. The surfaces containing >35 individual cells were analyzed. **(B)** IL-7Rα^high^ EM CD8^+^ T cells were treated with either TRAM-34 (5 µM) or margatoxin (50 nM), and the motility of the drug-treated cells on ICAM-1 and/or SDF-1α-coated flat PUA surfaces was recorded by time-lapse microscopy. The surfaces containing >20 individual cells were analyzed. **(C)** The migration of IL-2-reversed IL-7Rα^high^ and IL-7Rα^low^ EM CD8^+^ T cells was analyzed on ICAM-1 and/or SDF-1α-coated flat PUA surfaces. The results are representative data from two independent experiments using two different donors. Bars represent the mean, and *p*-values were obtained using the unpaired two-tailed Student’s *t*-test.

We examined the migration of IL-7Rα^low^ EM CD8^+^ T cells that had negligible levels of KCa3.1 activity. The motility of CD8^+^ T cells was determined on surfaces coated with ICAM-1 in the presence or absence of chemokine SDF-1α, which enhances motility of lymphocytes by inducing actin polymerization (26). After isolating EM CD8^+^ T cell subsets without activation, we assessed their migration by performing time-lapse microscopy and observed that IL-7Rα^high^ EM CD8^+^ T cells showed markedly higher mean velocity (*V*_mean_) than IL-7Rα^low^ EM CD8^+^ T cells (Figure 3A). Although SDF-1α modestly increased motility in both IL-7Rα^high^ and IL-7Rα^low^ EM CD8^+^ T cells, IL-7Rα^high^ EM CD8^+^ T cells showed substantially higher motility than IL-7Rα^low^ EM CD8^+^ T cells (Figure 3A). In line with these results, the TRAM-34 KCa3.1 inhibitor significantly inhibited the motility of IL-7Rα^high^ EM CD8^+^ T cells on ICAM-1 surfaces, even in the presence of SDF-1α (Figure 3B). Interestingly, the margatoxin Kv1.3 inhibitor did not affect T cell motility on the ICAM-1 surface; surprisingly, it increased motility in the presence of additional SDF-1α (Figure 3B).

Consistent with the recovery of KCa3.1 activity by IL-2 reversal (Figure 1D), the IL-2-reversed IL-7Rα^low^ EM CD8^+^ T cells showed similar levels of migration activity to those of IL-7Rα^high^ EM CD8^+^ T cells (Figure 3C). These data suggest that KCa3.1, but not Kv1.3, regulates the motility of IL-7Rα^low^ EM CD8^+^ T cells in the absence of TCR stimulation.

### Decreased Transendothelial Migration of IL-7Rα^low^ EM CD8^+^ T Cells Compared to That of IL-7Rα^high^ EM CD8^+^ T Cells

**Figure 4:**
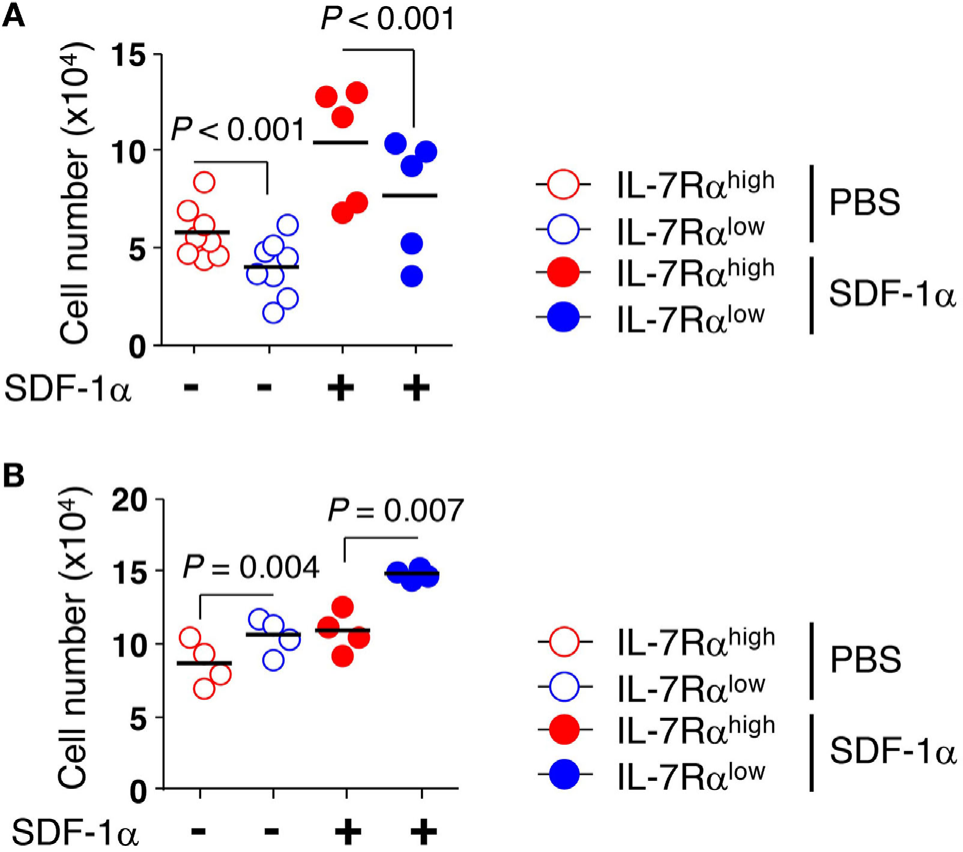
Decreased transendothelial migration of interleukin (IL)-7Rα^low^ effector memory (EM) CD8^+^ T cells compared to that of IL-7Rα^high^ EM CD8^+^ T cells. **(A)** freshly isolated or **(B)** IL-2-reversed IL-7Rα^high^ and IL-7Rα^low^ EM CD8^+^ T cells were added on top of human umbilical vein endothelial cell monolayers on the filters in the presence or absence of stromal cell-derived factor (SDF)-1α (100 ng/mL). Cells were allowed to migrate into the lower chambers or underneath the upper transwells for 20 h. Data are expressed as the number of cells that migrated across the filter. Results are representative data from two independent experiments from five to eight different donors. Bars represent the mean, and *p*-values were obtained using the unpaired two-tailed Student’s *t*-test.

We next examined whether the KCa3.1 channel activity of EM CD8^+^ T cells affected transendothelial migration. We utilized transwell chambers to measure T cell migration across a HUVEC monolayer, mimicking the *in vivo* microenvironment for T cell migration into local tissues. EM CD8^+^ T cell subsets were allowed to migrate through a HUVEC monolayer in the presence or absence of SDF-1α in the lower chamber. The transendothelial migration was significantly higher in IL-7Rα^high^ EM CD8^+^ T cells than in IL-7Rα^low^ EM CD8^+^ T cells. The low level of transendothelial migration of IL-7Rα^low^ EM CD8^+^ T cells was enhanced by adding SDF-1α (Figure 4A). Furthermore, IL-2 reversal restored the decreased transendothelial migration of IL-7Rα^low^ EM CD8^+^ T cells to a level surpassing even that of IL-7Rα^high^ EM CD8^+^ T cells (Figure 4B). However, in contrast to a previous result (Figure 3B), the KCa3.1 inhibitor failed to suppress transendothelial migration of IL-7Rα^high^ EM CD8^+^ T cells and IL-2-reversed EM CD8^+^ T cell subsets (data not shown). We postulate that an unknown mechanism, other than the KCa3.1 channel, might facilitate the complex process of transendothelial migration.

### IL-2 and IL-15 Increased KCa3.1 Channel Activity and the Motility of IL-7Rα^low^ EM CD8^+^ T Cells

**Figure 5:**
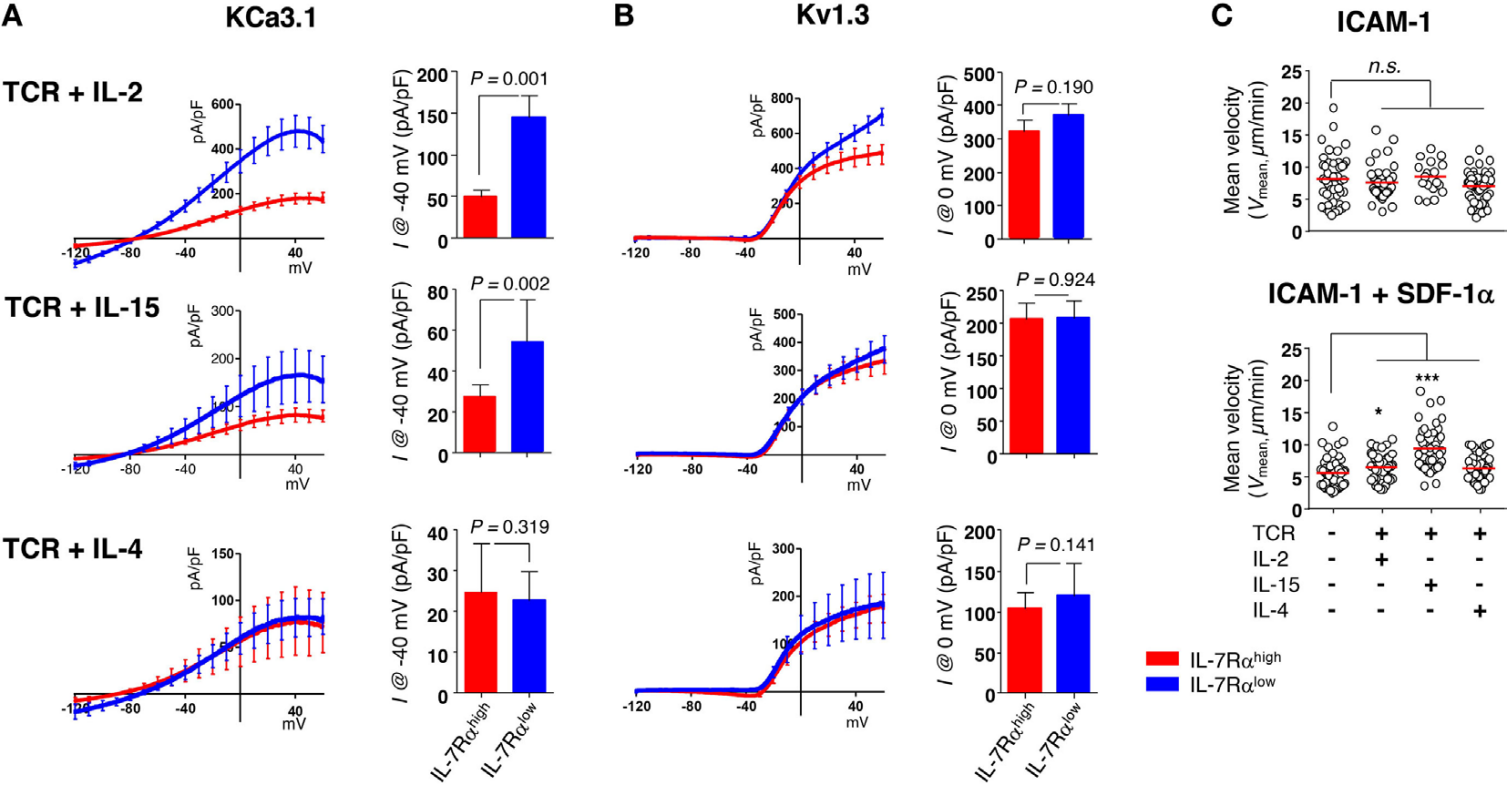
Interleukin (IL)-2 and IL-15 stimulation control the KCa3.1 activity in IL-7Rα^low^ effector memory (EM) CD8^+^ T cells. **(A,B)** To measure the current components of cytokine-stimulated IL-7Rα^high^ and IL-7Rα^low^ EM CD8^+^ T cells, cells were stimulated for 3 days with anti-CD3/CD28 antibodies in the presence of IL-2 (20 IU/mL), IL-15 (5 ng/mL), or IL-4 (5 ng/mL), and their KCa3.1 **(A)** and Kv1.3 **(B)** current components (IL-7Rα^high^ versus IL-7Rα^low^: *n* = 17 versus *n* = 15; *n* = 8 versus *n* = 7; or *n* = 6 versus *n* = 7, respectively) were measured as described in Figure 1. Bars and error bars represent the mean ± SEM, and *p*-values were obtained using the unpaired two-tailed Student’s *t*-test. **(C)** The migration of cytokine-stimulated IL-7Rα^low^ EM CD8^+^ T cells was analyzed as described in Figure 2. The results are representative data from two independent experiments using two different donors. Bars represent the mean, and *p*-values were obtained using the unpaired two-tailed Student’s *t*-test.

According to our data published previously (13, 27), IL-2 and IL-15 are both capable of reviving the impaired proliferative function of IL-7Rα^low^ EM CD8^+^ T cells (Sim et al., manuscript submitted). Thus, we analyzed the KCa3.1 activity and motility in T cells stimulated for 3 days with anti-CD3/CD28 Abs in the presence of IL-2, IL-15, or IL-4 (i.e., short-term cytokine stimulation). Short-term cytokine stimulation with IL-2 (IL-7Rα^high^ versus IL-7Rα^low^, 49.26 ± 8.28 versus 144.51 ± 26.15 pA/pF) or IL-15 (27.37 ± 5.56 versus 54.27 ± 20.50 pA/pF), but not IL-4 (24.76 ± 11.83 versus 22.63 ± 7.22 pA/pF), increased the KCa3.1 activity considerably in IL-7Rα^low^ EM CD8^+^ T cells (Figure 5A). However, the cytokine stimulation had no effect on Kv1.3 activity (Figure 5B). But, as with the above results (Figure S2A in Supplementary Material), expression levels of Kv1.3 and KCa3.1 transcript did not reflect both of the potassium channel activities in IL-7Rα^high^ and IL-7Rα^low^ EM CD8^+^ T cells even under the cytokine-stimulated condition (Figure S2B in Supplementary Material). Likely for the same reason, the expression of functional potassium channel seems to be regulated by posttranslational regulation. Although neither IL-2 nor IL-15 affected the motility of IL-7Rα^low^ EM CD8^+^ T cells on the ICAM-1 surface, both cytokines increased the motility markedly in the presence of SDF-1α (Figure 5C). This result suggests that the functionally revived IL-7Rα^low^ EM CD8^+^ T cells by short-term stimulation with IL-2 or IL-15 regained motility in the presence of SDF-1α due to increased KCa3.1 activity. Our findings suggest that T cells activated with TCR stimulation and inflammatory cytokines could revive cell motility *via* increasing KCa3.1 expression, eventually leading to the accumulation of innately motility-impaired IL-7Rα^low^ EM CD8^+^ T cells in the inflammatory site.

### Dominant Skin-Infiltrating CD8^+^ T Cells in Healthy Tissues Are CD8^+^ T Cells Expressing High Levels of IL-7Rα and Low Levels of Perforin

Next, we hypothesized that IL-7Rα^high^ EM CD8^+^ T cells, which have significant KCa3.1 activity, are more frequently present in tissue than IL-7Rα^low^ EM CD8^+^ T cells are because of their excellent *in vitro* migration activity. Thus, we analyzed the tissue infiltration of EM CD8^+^ T cell subsets in non-lesional (healthy, HC) and lesional (dermatitis) skin. Our previous finding showed that IL-7Rα^low^ EM CD8^+^ T cells are generally found to be largely perforin^high^ compared to IL-7Rα^high^ EM CD8^+^ T cells (13). Moreover, IL-15 stimulation increases intracellular perforin expression (28), suggesting that CD8^+^ T cells could increase perforin expression in inflammatory conditions. Thus, we performed immunofluorescence staining on skin-infiltrating IL-7Rα^high^ and IL-7Rα^low^ EM CD8^+^ T cells using an Ab combination for IL-7Rα and CD8, or perforin and CD8, respectively.

**Figure 6:**
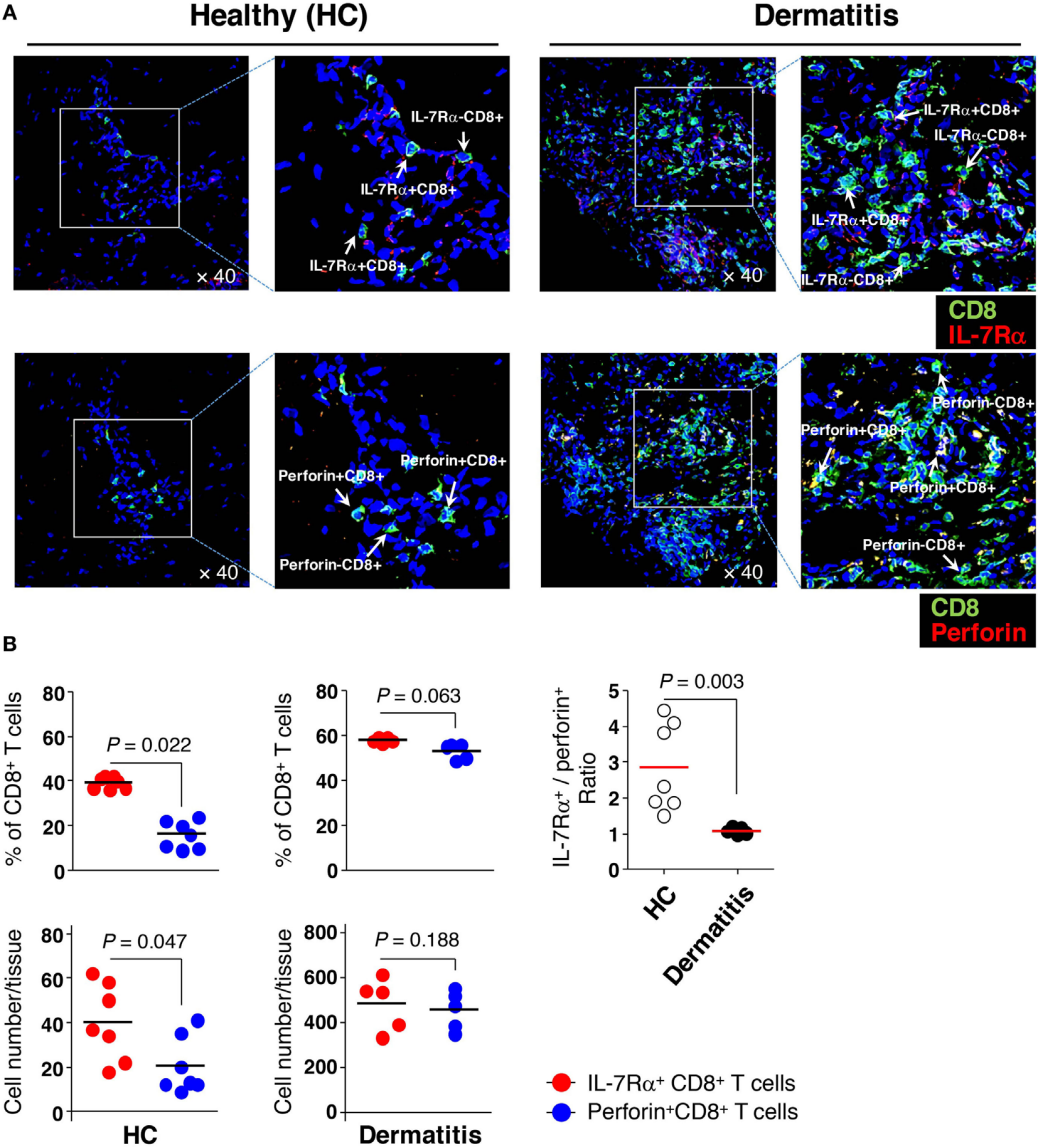
Greater numbers of interleukin (IL)-7Rα^high^ effector memory (EM) CD8^+^ T cells than IL-7Rα^low^ EM CD8^+^ T cells in the skin. **(A)** Immunofluorescence staining (40×) of CD8^+^ T cells (green) from non-lesional (healthy, HC) or lesional atopic dermatitis skin. IL-7Rα^+^ CD8^+^ T cells (upper panel) representing IL-7Rα^high^ EM CD8^+^ T cells were stained with antibodies (Abs) to IL-7Rα (red); perforin^+^ CD8^+^ T cells (lower panel) representing IL-7Rα^low^ EM CD8^+^ T cells, were stained with Abs to perforin (red). The image of the box was magnified twice and placed to the right of each image. **(B)** A quantitative measurement of IL-7Rα^+^ and perforin^+^ CD8^+^ T cells (frequency and number per tissue) in panel **(A)**, representing IL-7Rα^high^ and IL-7Rα^low^ EM CD8^+^ T cells, respectively. Four images per slide were evaluated for quantification. Data are representative of four independent experiments. Bars represent the mean, and *p*-values were obtained using the Wilcoxon matched pairs test (for comparing frequency and number between the two CD8^+^ T cell subsets) and Mann–Whitney *U* test (for comparing IL-7Rα^+^/perforin^+^ ratio between HC and dermatitis).

In the non-lesional HC tissues, the number and frequency of IL-7Rα^+^ CD8^+^ T cells reflecting IL-7Rα^high^ EM CD8^+^ T cells were significantly higher than the number and frequency of perforin^+^ CD8^+^ T cells that correspond to IL-7Rα^low^ EM CD8^+^ T cells (Figures 6A,B). Meanwhile, in the lesional (dermatitis) tissue, both IL-7Rα^+^ and perforin^+^ CD8^+^ T cells were increased (Figures 6A,B). However, the ratio of IL-7Rα^+^ CD8^+^ T cells to perforin^+^ CD8^+^ T cells in non-lesional HC tissue was higher than that in the lesional tissue (Figure 6B), indicating that IL-7Rα^high^ EM CD8^+^ T cells comprised the majority of the population in non-inflammatory sterile tissue.

These results suggest that IL-7Rα^high^ EM CD8^+^ T cells may preferentially reside in non-inflammatory sterile tissue and that increased perforin expression in lesional tissue may be secondary to either the acquisition of perforin molecules by IL-7Rα^+^ CD8^+^ T cells or the enhanced migration of perforin^+^ CD8^+^ T cells by inflammatory stimuli such as cytokines, as supported by previous data (Figure 5C).

## Discussion

This study demonstrated that the potassium channels Kv1.3 and KCa3.1 are differentially expressed in functionally distinct IL-7Rα^high^ and IL-7Rα^low^ EM CD8^+^ T cells. IL-7Rα^low^ EM CD8^+^ T cells showed lower activity of Kv1.3 and weak conductance of KCa3.1 (Figure 1A). The low Kv1.3 activity reflecting IL-7Rα^low^ EM CD8^+^ T cells might partly underlie the impairment in proliferation and IL-2 production in response to TCR stimulation. Interestingly, IL-2 reversal induces IL-7Rα^low^ EM CD8^+^ T cells to become functionally competent cells exhibiting potassium channel activity levels comparable to IL-7Rα^high^ EM CD8^+^ T cells (Figure 1D). These results suggest that the potassium channel activity in human EM CD8^+^ T cells may be associated with the function of the cells.

Consistent with the critical role of KCa3.1 for the motility of different types of cells [reviewed in Ref. (23)], our present study shows that motility on an ICAM-1 surface was significantly decreased in IL-7Rα^low^ EM CD8^+^ T cells that had insignificant KCa3.1 activity (Figure 3A). Furthermore, the lower motility of IL-7Rα^low^ EM CD8^+^ T cells was revived by several treatments, including IL-2, which caused full recovery of IL-7Rα^low^ CD8^+^ T cell function, or short-term IL-2 and IL-15 stimulation, which caused an increase in KCa3.1 current (Figures 1D and 5). Finally, the motility of IL-7Rα^high^ EM CD8^+^ T cells on the ICAM-1 surface was significantly repressed by the KCa3.1 inhibitor, but not by the Kv1.3 inhibitor (Figure 3B). In a transendothelial migration analysis performed using a HUVEC monolayer, we found that transendothelial migration of IL-7Rα^low^ EM CD8^+^ T cells was reduced significantly compared to that of IL-7Rα^high^ EM CD8^+^ T cells (Figure 4A), but could be restored by IL-2 reversal (Figure 4B). The finding that short-term IL-2 and IL-15 stimulation induced KCa3.1 activity and cell motility suggests that this inflammatory cue may enhance cell motility *via* upregulation of the KCa3.1 in EM CD8^+^ T cells. In sterile skin, the number and frequency of IL-7Rα^+^ CD8^+^ T cells, which reflect the number and frequency of IL-7Rα^high^ EM CD8^+^ T cells in non-lesional tissue, were significantly higher compared to those found in perforin^+^ CD8^+^ T cells that correspond to IL-7Rα^low^ EM CD8^+^ T cells (Figures 6A,B). This result suggests that IL-7Rα^high^ EM CD8^+^ T cells in healthy tissues might constitute the majority of the population owing to their intrinsic migratory capability.

Here, we demonstrated that the proliferation and IL-2 production of human EM CD8^+^ T cells without prior stimulation were modestly regulated by Kv1.3, but not KCa3.1 (Figures 2A,B). These results differ from previous studies of pre-activated T cells, although the cells used in previous studied are different from our EM CD8^+^ T-cell subsets, which showed that KCa3.1 contributes to pre-activated T cell proliferation (29) and cytokine release (25). Interestingly, adenosine was used to inhibit KCa3.1 *via* the A_2A_R receptor in pre-activated T cells for more than 72 h with TCR stimulation, resulting in the inhibition of IL-2 production (25). In our study, however, KCa3.1 inhibition exerted no influence on the production of IL-2 or other cytokines, such as IFN-γ or TNF-α (Figure 2B). Such variations among studies might be due to a number of differences including the pre-stimulation conditions or the use of a specific subset of T cells. First, concerning the differences in T cell activation status, we used freshly sorted EM CD8^+^ T cells, whereas the previous study used phorbol myristoyl acetate/ionomycin- or anti-CD3/CD28 Ab-stimulated T cells for 72−96 h (29). Second, the adenosine-induced IL-2 suppression in T cells may be mediated by mechanism(s) other than the KCa3.1 channel. For example, Sevigny et al. (30) reported that ATL313, a selective A_2A_R agonist, inhibits T cell activation, proliferation, and cytokine production by the cAMP/protein kinase A (PKA) pathway. Although the KCa3.1 current is inhibited by PKA through phosphorylation and consequent decrease in calmodulin binding (31), PKA in T cells also phosphorylates nuclear factor of activated T cells (NFAT) and inhibits NFAT transcriptional activity that is critical for T cell activation (32). Finally, considering that CD4^+^ T cells from KCa3.1 knockout mice are defective in the production of IL-2, IFN-γ, and TNF-α, whereas Th17 and regulatory T cell (Treg) functions are normal following TCR stimulation (33), there may be species- or cell type-specific responses to the KCa3.1 channel.

This study demonstrated the requirement of Kv1.3 activity for IL-7Rα^high^ EM CD8^+^ T cell proliferation and production of IL-2, IFN-γ, and TNF-α (Figures 2A,B). Other studies also reported that a selective Kv1.3 inhibitor (SL5) inhibits proliferation as well as IL-2 and IFN-γ production in T cells from synovial fluid (mainly EM cells). In contrast, such an effect was less significant in the peripheral blood T cells (mainly naïve/central memory) from patients with rheumatoid arthritis; these inhibitors are less effective in suppressing the production of TNF-α and IL-4 (34). Thus, the differing sensitivities of T cells to Kv1.3 blockade seems to be due to the different subsets and antigen experience of the investigated T cells. In this context, Chiang et al. (8) showed that repeatedly stimulated antigen-specific T cells become “programmed” toward Kv1.3 dependency. This is consistent with our result that Kv1.3 inhibitor, not the KCa3.1 inhibitor, regulates the proliferation and IFN-γ production of IL-7Rα^high^ EM CD8^+^ T cells under the condition where KCa3.1 activity is functional (Figures 1 and 2). Importantly, the characteristics of antigen-specific cells suggested by Chiang et al. were similar to those of IL-7Rα^high^ EM CD8^+^ T cells in terms of potassium channel activity, although the extent of prior antigenic experience is lower than with IL-7Rα^low^ EM CD8^+^ T cells.

Our findings demonstrated that IL-7Rα^high^ EM CD8^+^ T cells were the predominant CD8^+^ T cell subsets in sterile skin (Figures 6A,B), which was extrapolated from the fact that IL-7Rα^high^ EM CD8^+^ T cells demonstrated increased motility, particularly in transendothelial migration capability, compared to IL-7Rα^low^ EM CD8^+^ T cells (Figures 1, 3 and 4). Given that IL-15 is abundant in inflammatory conditions (28), and that IL-15 revives the motility of IL-7Rα^low^ EM CD8^+^ T cells, our findings indicate that cytokine stimulation may play a role in the regulation of KCa3.1. Although IL-7Rα^low^ EM CD8^+^ T cells had defects in T cell proliferation upon TCR stimulation, these cells had higher levels of cytotoxic molecules such as perforin, granzyme B, and 2B4, possibly contributing to tissue damage through 2B4-mediated cytotoxicity (14). Thus, the human immune system is thought to have developed in a direction that avoids excessive tissue damage: the system represses the migration of cells into local tissues until the inflammation becomes excessive, at which point the invasion of bacteria or virus from the external environment will allow it to produce inflammatory cytokines, such as IL-2 and IL-15. This idea is supported by the finding that perforin^+^ CD8^+^ T cells, which represent IL-7Rα^low^ EM CD8^+^ T cells, were increased in dermatitis affected, rather than IL-7Rα^+^ CD8^+^ T cells (Figure 6B). This result is consistent with the report by Shin et al., which showed that IL-7Rα^low^ EM CD8^+^ T cells having high expression of CX_3_CR1 demonstrate increased migration capacity in response to fractalkine. This result suggests that CX_3_CR1-expressing IL-7Rα^low^ EM CD8^+^ T cells may move into the infected or damaged peripheral site in response to fractalkine (35). In addition, CX_3_CR1^−^ CD8^+^ T cells expressing intermediate levels of IL-2 and low levels of granzyme B are a source for tissue-resident memory in epithelial tissue, presumably providing local tissue protection in a manner independent of cytotoxicity by IFN-γ-mediated induction of anti-bacterial and anti-viral genes (36, 37).

Kv1.3 contributed to CD8^+^ T cell proliferation and IL-2 and TNF-α production, but not IFN-γ production, whereas KCa3.1 supported the cell migration of CD8^+^ T cells. These findings contribute to our understanding of how EM CD8^+^ T cells from healthy individuals work differently based on their potassium channel expression. Furthermore, our data demonstrate that reduced potassium channel activities can be revived by cytokines, eventually leading to the restoration of impaired IL-7Rα^low^ EM CD8^+^ T cell function. Although further research is needed, our data suggest the possible scenario that functional CD8^+^ T cells (e.g., IL-7Rα^high^ EM CD8^+^ T cells with intact potassium channels) may function as a reservoir for effector CD8^+^ T cells, helping to clear out pathogens or control inflammation in the local tissue.

## Ethics Statement

This work was approved by the Institutional Review Board of Seoul National University Hospital (# 0905-014-280). Peripheral blood was obtained from healthy volunteers who were taking no immunosuppressive drugs and had no diseases that could potentially affect the immune system such as autoimmunity, infections, and malignancies. Skin specimens (5 mm diameter) were obtained from a patient who had moderate atopic dermatitis (AD) with chronic lesional and non-lesional skin. Written informed consent was obtained from all subjects according to the Declaration of Helsinki.

## Author Contributions

H-RK, JD, C-HC, and SK had full access to all data in the study and took responsibility for the integrity of the data, as well as for the manuscript. JS, KK, HP, K-JK, and H-RK performed most of the experiments, data analysis, and manuscript preparation. HL, T-JK, HS, GK, D-SL, and DL participated in data acquisition and analysis. All the authors have read and approved the final manuscript.

## Conflict of Interest Statement

The authors declare that the research was conducted in the absence of any commercial or financial relationships that could be construed as a potential conflict of interest.

## Abbreviations

IL-7Rα, IL-7 receptor alpha (α)-chain; PBMCs, peripheral blood mononuclear cells; EM, effector memory; HUVECs, human umbilical vein endothelial cells; ICAM-1, intercellular adhesion molecule 1; SDF-1α, stromal cell-derived factor-1α.
