# Supplementary material for: Differentially Expressed Potassium Channels Are Associated with Function of Human Effector Memory CD8+ T Cells
Source: Front Immunol. 2017 Jul 24;8:859. doi: 10.3389/fimmu.2017.00859 (PMC5522836; doi:10.3389/fimmu.2017.00859)
Supplement: Supplementary file 5 [file data_sheet_1.docx]

Supplementary Movie legends

Movie S1. A representative movie of migrating IL-7Rα^high^ EM CD8^+^ T cells on a surface coated with ICAM-1 acquired by differential interference contrast (DIC) microscopy. Scale bar: 10 µm. Elapsed time: mm:ss.

Movie S2. A representative movie of migrating IL-7Rα^low^ EM CD8^+^ T cells on a surface coated with ICAM-1 acquired by DIC microscopy. Scale bar: 10 µm. Elapsed time: mm:ss.

Movie S3. A representative movie of migrating IL-7Rα^high^ EM CD8^+^ T cells on a surface coated with ICAM-1 and SDF-1α acquired by DIC microscopy. Scale bar: 10 µm. Elapsed time: mm ss.

Movie S4. A representative movie of migrating IL-7Rα^low^ EM CD8^+^ T cells on a surface coated with ICAM-1 and SDF-1α acquired by DIC microscopy. Scale bar: 10 µm. Elapsed time: mm:ss.
