# Supplementary material for: Differentially Expressed Potassium Channels Are Associated with Function of Human Effector Memory CD8+ T Cells
Source: Front Immunol. 2017 Jul 24;8:859. doi: 10.3389/fimmu.2017.00859 (PMC5522836; doi:10.3389/fimmu.2017.00859)
Supplement: Supplementary file 6 [file data_sheet_2.pdf]

SUPPLEMENTARY MATERIALS AND METHODS

Intracellular Ca^{2+} Measurements

$CD8^+$ T-cell subsets were loaded with 1 µM Fura-2/AM (Invitrogen, Carlsbad, CA, USA) for 30 min at 37 °C in Ca^{2+}-free HSBB medium, washed twice with R10 media, and attached to poly-L-lysine treated glass cover slips loaded in a Chamlide magnetic chamber (Live Cell Instrument). The Fura-2/AM loaded cells were stimulated with anti-CD3 (OKT3, 5 µg/mL, eBioscience) and thapsigargin (2 µM, Sigma Aldrich), and then were monitored on a Zeiss Axio Observer.Z1 epifluorescence microscope. Fura-2-340 (EX BP 340/30, EM BP 510/90) and Fura-2-380 (EX BP 387/15, EM BP 510/90) excitation/emission filters sets were used for fluorescence imaging. Intracellular calcium was imaged for 15 min with 5 s intervals and Fura-2 340/380 ratio images were analyzed with Metamorph (Molecular Devices).

Quantitative RT-PCR

Total RNA was isolated from freshly sorted and cytokine-stimulated $CD8^+$ T cell subsets using RNeasy mini prep kit (Qiagen, Valencia, CA, USA) and cDNA synthesis was generated by Transcriptor First Strand cDNA Synthesis Kit (Roche Applied Science, Indianapolis, IN, USA). Polymerase chain reaction (PCR) was performed with a CFX Connect™ Real-Time PCR Detection System using an iQ™ SYBR® Green Supermix (Bio-Rad, Hercules, CA, USA). All results were normalized to expression of the control gene *RPS18* (encoding Ribosomal Protein S18). Primers used in real-time PCR were as follows: *KCNA3* (Kv1.3) forward, 5′- CCA-GTA-CAT-GCA-CGT-GGG-AA-3′, and reverse, 5′-GCA-GTG-GAA-TTG-CCC-GTT-TT-3′; *KCNN4* (KCa3.1) forward, 5′- ATC-GGC-GCT-CTC-AAT-CAA-GT-3′, and reverse, 5′- ATT-AAC-AGC-CTG-CCT-CTC-GG-3′; *RPS18* forward, 5′-GAT-GGG-CGG-CGG-AAA-ATA-G-3′, and reverse, 5′- GCG-TGG-ATT-CTG-CAT-AAT-GG-3′.
