# Supplementary material for: Differentially Expressed Potassium Channels Are Associated with Function of Human Effector Memory CD8+ T Cells
Source: Front Immunol. 2017 Jul 24;8:859. doi: 10.3389/fimmu.2017.00859 (PMC5522836; doi:10.3389/fimmu.2017.00859)
Supplement: Supplementary file 7 [file data_sheet_3.pdf]

SUPPLEMENTARY FIGURE LEGENDS

Figure S1. Calcium influx of EM CD8$^+$ T cell subsets differentially expressing Kv1.3 and KCa3.1. Intracellular [Ca^{2+}] in Fura-2 AM-loaded IL-7Rα^{high} (n = 9) and IL-7Rα^{low} (n = 7) EM CD8$^+$ T cells stimulated with anti-CD3 (OKT3 clone) Abs or thapsigargin (TG) were determined using fluorescence microscopy. Intracellular [Ca^{2+}] level was presented as the ratio of emitted fluorescence (510 nm) after excitation at 340 and 380 nm. The fluorescence intensity ratio was normalized to the ratio of resting state prior to cell stimulation (Fura-2 340/380 ratio). The results are representative data from three independent experiments. Lines represent the mean.

Figure S2. Quantitative RT-PCR analysis of Kv1.3 and KCa3.1 for CD8$^+$ T cell subsets. (A) Freshly sorted and (B) cytokine-stimulated IL-7Rα^{high} and IL-7Rα^{low} EM CD8$^+$ T cells were subjected to quantitative RT-PCR to determine the relative gene expression of Kv1.3 and KCa3.1. For cytokine-stimulated IL-7Rα^{high} and IL-7Rα^{low} EM CD8$^+$ T cells, cells were stimulated for 3 days with anti-CD3/CD28 Abs in the presence of either IL-2 (20 IU/mL), IL-15 (5 ng/mL), or IL-4 (5 ng/mL). *P*-values were obtained using the paired two-tailed Student's *t* test. Results are representative data from two independent experiments (n = 5).
