# Supplementary figures and images for: Differentially Expressed Potassium Channels Are Associated with Function of Human Effector Memory CD8+ T Cells
Source: Front Immunol. 2017 Jul 24;8:859. doi: 10.3389/fimmu.2017.00859 (PMC5522836; doi:10.3389/fimmu.2017.00859)

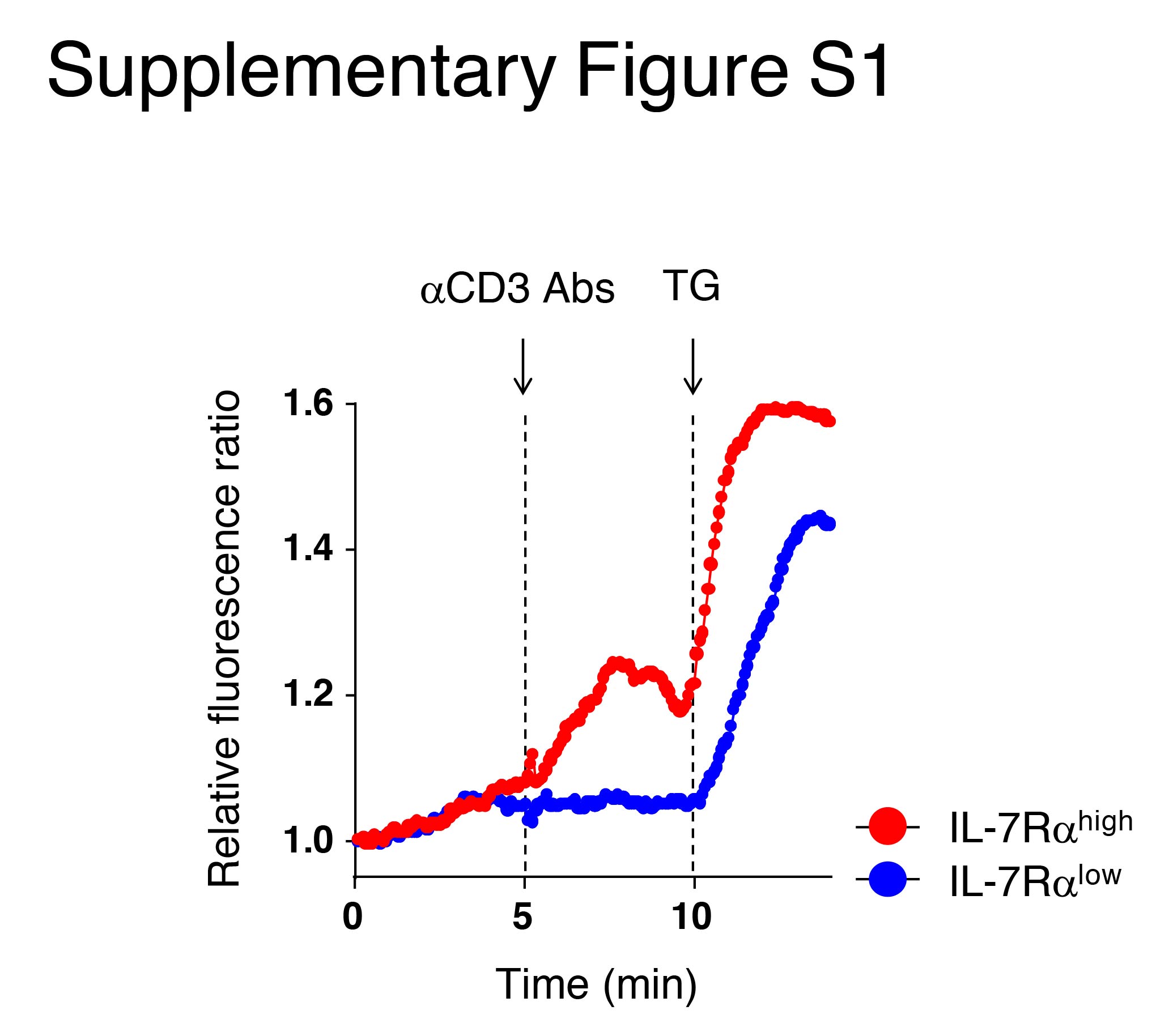

Supplement: Supplementary file 8 [file image_1.jpeg]

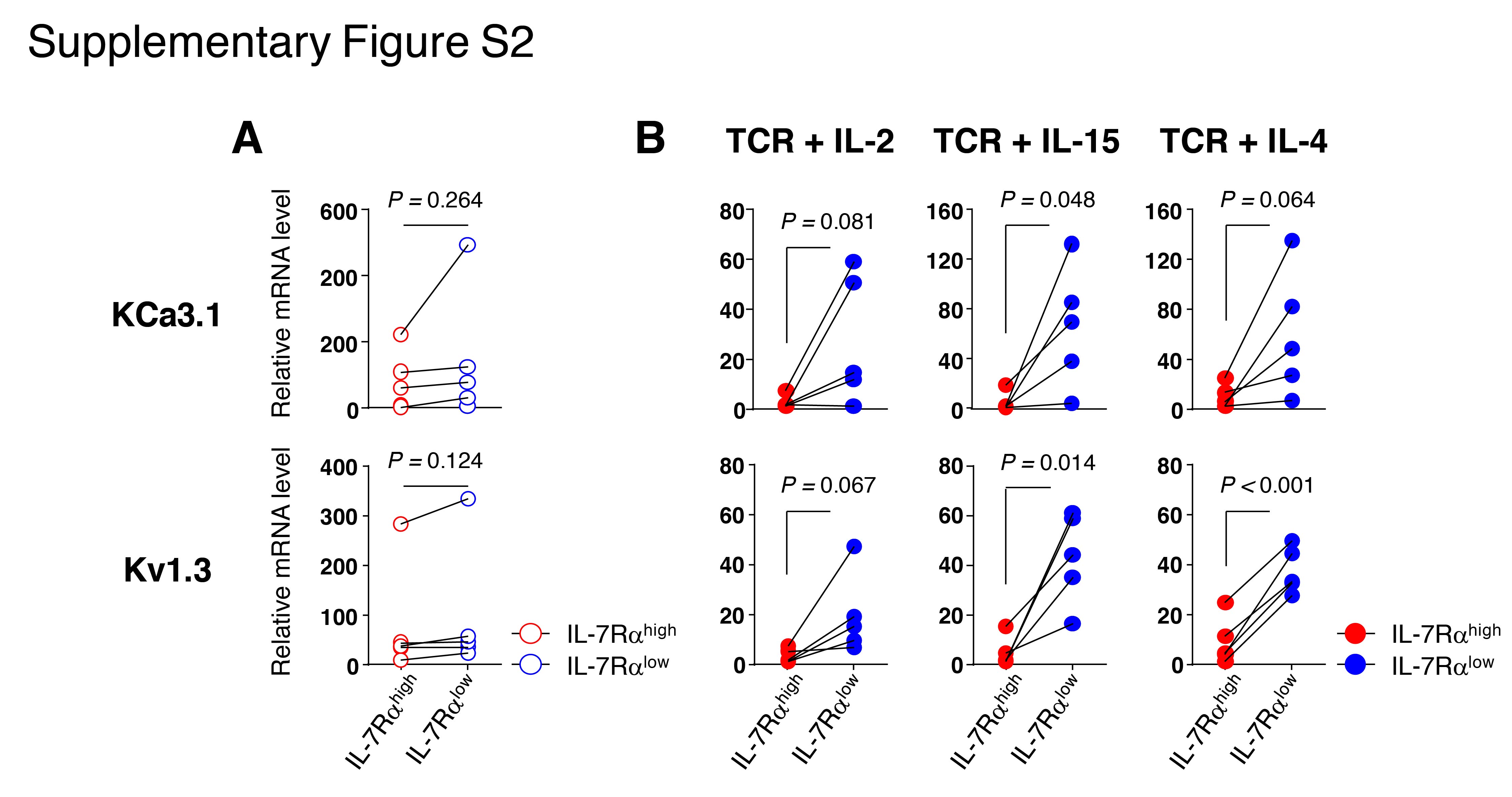

Supplement: Supplementary file 9 [file image_2.jpeg]
